# Genome-wide identification, expression, and regulatory network analysis of wheat microRNAs responsive to *Bipolaris sorokiniana*

**DOI:** 10.3389/fpls.2025.1640327

**Published:** 2025-11-27

**Authors:** Natasha Kashyap, Malkhan Singh Gurjar, Poulami Basak, Xizhe Sun, Lisong Ma, Aundy Kumar, Jyoti Kumari, Rashmi Aggarwal, Mahender Singh Saharan, Sambasivam Periyannan

**Affiliations:** 1 Division of Plant Pathology, Indian Agricultural Research Institute, Indian Council of Agricultural Research, New Delhi, India; 2 State Key Laboratory of North China Crop Improvement and Regulation, Hebei Agricultural University, Baoding, China; 3 Indian Council of Agricultural Research, National Bureau of Plant Genetic Resources, New Delhi, India; 4 Centre for Crop Health, University of Southern Queensland, Toowoomba, QLD, Australia; 5 School of Agriculture and Environmental Science, University of Southern Queensland, Toowoomba, QLD, Australia

**Keywords:** wheat, spot blotch, *Bipolaris sorokiniana*, miRNA, defense, host-pathogen interaction

## Abstract

Spot blotch, caused by *Bipolaris sorokiniana*, is an important disease that leads to significant economic losses in wheat globally. Due to the complexity of *B. sorokiniana* infection, identification of wheat lines with strong resistance to spot blotch is challenging. Hence, the introduction of effective disease management strategies through the manipulation of genes involved in *B. sorokiniana*–wheat interaction remains essential. MicroRNAs (miRNAs) play a vital role in gene regulation and are increasingly used to predict molecular networks and genes associated with disease development or resistance. In this study, we employed small RNA sequencing to profile miRNAs in a resistant (IC566637) and a susceptible (Agra Local) wheat genotype following *B. sorokiniana* infection. A total of 726 miRNAs, predominantly 21 to 22 nucleotides in length, were identified. Among these, 140 are differentially expressed (DE) and associated with the modulation of 894 genes. The Gene Ontology (GO) and Kyoto Encyclopedia of Genes and Genomes (KEGG) enrichment analysis revealed these target genes as secondary metabolites, ATB-binding cassette (ABC) transporters, nucleotide-binding leucine-rich repeat (NB-LRR), mitogen-activated protein kinase (MAPK) genes, and hormones associated with plant–pathogen interaction and defense signal transduction. The regulatory network constructed from this data highlights key miRNA–target interactions likely contributing to disease resistance. Quantitative RT-PCR validation of nine selected miRNAs and their corresponding target genes further supports their potential role in modulating wheat defense responses. These findings provide a comprehensive resource for understanding miRNA-mediated regulation in the wheat–*B. sorokiniana* pathosystem and identified promising candidate genes for future resistance breeding and genome editing efforts.

## 1 Introduction

Spot blotch, caused by *Bipolaris sorokiniana* (Sacc.), is among the most dreadful diseases of wheat worldwide and a serious issue in Southeast Asia due to hot and humid weather conditions (Chaurasia et al., 2000; Chand et al., 2008; Vaish et al., 2011; Al-Sadi, 2016). In India, although the disease was first reported in 1914, its incidence became more frequent after the green revolution, as the introduction of dwarf varieties with narrow genetic diversity aggravated the disease both domestically as well as in its neighboring countries such as China, Bangladesh, and Nepal (Singh et al., 2016; Gupta et al., 2018). Other than wheat, *B. sorokiniana* is also responsible for root rot and leaf spot diseases in other cereals such as oat, barley, maize, and rice (Devi et al., 2018; Basak et al., 2024).

In wheat, yield losses due to spot blotch disease range from 15%–25% and can lead to total crop loss in severe epidemic conditions (Sahu et al., 2016). The present wheat varieties lack sufficient resistance to this disease due to the pathogen’s hemibiotrophic nature, high population diversity, and its ability to alter the expression of host defense genes (Aggarwal et al., 2009; Gurjar et al., 2018; Singh et al., 2018; Tembo et al., 2018; Aggarwal et al., 2019). Understanding the molecular basis of host–pathogen interactions is essential to develop durable resistance and informed breeding strategies.

MicroRNAs (miRNAs) are small non-coding RNAs that play key roles in regulating gene expression during plant development and stress responses, including biotic stresses caused by pathogens. These molecules also regulate immune signaling pathways during pathogen infection and disease development (Islam et al., 2018; Šečić et al., 2021). For instance, ath-miR393, induced by flg22, was the first identified miRNA associated with plant immunity, conferring resistance to *Pseudomonas syringae* pv. *tomato* by targeting receptors in auxin signaling (Navarro et al., 2006). Other miRNAs, such as ath-miR398b, ath-miR160a, and ath-miR773, have been associated with the regulation of pattern-triggered immunity (PTI) and cell-wall-associated defense responses in *Arabidopsis* (Li et al., 2010). In rice, overexpression of miR164a, targeting NAC (NAM, ATAF1/2, and CUC2) transcription factor, was linked with susceptibility to *Magnaporthe oryzae* (Wang et al., 2018). Notably, this regulatory module is conserved for various crop diseases, including sheath blight, late blight, and root/stem rot diseases in rice, tomato, and soybean, respectively.

In wheat, miR156, miR166, miR167, miR398, and miR399 were associated with resistance to leaf rust, while miR7723 promotes susceptibility (Kumar et al., 2016). Furthermore, the defense-related genes such as Leucine-rich repeat (LRR) receptor-like serine/threonine-protein kinase (targeted by bdi-miR167), Resistance to Peronospora parasitica (RPP)8 (targeted by TamiR007), and *Resistance to Pseudomonas maculicola (RPM)1*-like (targeted by TamiR138) were among the major types targeted by miRNAs (Kumar et al., 2016). However, the role of miRNAs in wheat defense against *B. sorokiniana* remains unclear due to technological limitations and the absence of a complete wheat reference genome. For instance, Inal et al. (2014) utilized microarray technology to identify miRNAs (miR164, miR169, miR319, and miR398) involved in the wheat–*B. sorokiniana* interaction but failed to detect significant differences in their expression in resistant and susceptible cultivars. Later, Sharma et al. (2021) reported only the miRNAs (ptc-miR169, ptc-miR1450, and tae-miR156) identified from other pathosystems.

More recently, Vasistha et al. (2025) used differentially expressed (DE) genes in *B. sorokiniana*-infected wheat genotypes to predict miRNAs; however, the absence of direct small RNA (sRNA) profiling limited the resolution and accuracy of miRNA identification in the study. In light of these gaps, our study adopts a comprehensive approach using sRNA sequencing to analyze the differential expression of miRNAs in resistant and susceptible wheat genotypes following *B. sorokiniana* infection. Furthermore, with the gold standard chromosomal-level wheat genome assembly, a more comprehensive chromosome-wise *in silico* miRNA prediction allows the discovery of both the novel and conserved miRNAs. This enables the construction of a complete “atlas” of miRNAs involved in *B. sorokiniana*–wheat interaction, providing a more robust and precise understanding of the gene regulatory networks in this host–pathogen interaction (Jaiswal et al., 2019).

Our analysis revealed a set of DE miRNAs associated with the regulation of key defense-related genes, including those involved in hormone signaling, secondary metabolism, ABC transporters, NB-LRR resistance proteins, and mitogen-activated protein kinase (MAPK) cascades. Further validation through stem loop qRT-PCR supported the involvement of selected miRNAs and their targets in modulating wheat’s response to infection. To our knowledge, this is the first study to provide a publicly available sRNA dataset specific to the wheat–*B. sorokiniana* interaction. These findings not only enhance our understanding of miRNA-mediated defense mechanisms in wheat but also identify promising candidate targets for future functional validation and genetic improvement efforts aimed at enhancing spot blotch resistance.

## 2 Materials and methods

### 2.1 Plant material and fungal inoculation

The spot-blotch-resistant wheat genotype IC566637 was obtained from the Indian Council of Agricultural Research (ICAR)—National Bureau of Plant Genetic Resources (NBPGR), New Delhi, India, while Agra Local served as the susceptible control. Seeds were sown in 4-inch. pots containing pre-sterilized soil and maintained at 25 ± 2 °C in the greenhouse at the Division of Plant Pathology, ICAR—Indian Agricultural Research Institute (IARI), New Delhi. A pure culture of *B. sorokiniana* isolate BS-22 (virulent on Agra Local, NCBI accession number ON892001, Indian Type Culture Collection identification number 9114), mass-cultured using potato dextrose agar medium and autoclaved sorghum grains (25–30 days), was used for the study. Seedlings at the three- to four-leaf stage were inoculated with the spore suspension (10^4^ conidia/mL) using a precision hand atomizer. Sterile distilled water was used as untreated control. To maintain high humidity (>90%), the plants were kept in a humidity chamber box for 24–48 h. Treatments consisted of susceptible inoculated (SI) and susceptible untreated control (SC) as well as resistant inoculated (RI) and resistant untreated control (RC). Leaf samples from inoculated and control plants were collected at 0, 12, 24, 36, 48, and 60 h post-inoculation in liquid nitrogen and stored in a deep freezer at -80°C until further use. Each sample consisted of three biological replicates.

### 2.2 RNA extraction, small RNA library preparation, and sequencing

For small RNA sequencing (sRNA-seq), two biological replicates per treatment were used, with samples pooled across time points. Total RNA was extracted from leaf tissue using the Direct-ZOL mini kit (Zymo Research) following standard protocols. RNA concentration and purity were measured using a Nanodrop spectrophotometer (Thermo Scientific, 2000). The proportion of miRNA in the samples were determined using a Bioanalyzer (Agilent), while RNA quantification was performed with the Qubit RNA HS assay kit (Thermo Scientific). QIAseq^®^ miRNA Library Kit (Qiagen) standard protocol was used to generate the sRNA-seq libraries. In brief, 63 ng of total RNA was used as initial input. The 3′ adapters were selectively ligated to the 3′OH group of miRNAs, followed by 5′ adapter ligation. The ligated fragments were subjected to reverse transcription with Unique Molecular Index (UMI) assignment by priming using reverse transcription primers. The resulting cDNA was then amplified and barcoded through PCR amplification (17 cycles). The Illumina-compatible sequencing libraries were quantified using the Qubit fluorometer (Thermo Fisher Scientific, MA, USA). Finally, single-end sequencing was performed for 50 cycles on Illumina NovaSeq 6000 High Output sequencing platform using SE50 read length and sequencing chemistry.

### 2.3 Sequencing data analysis, identification of known and novel miRNAs

Raw reads of 16–40 bases in length were filtered and mapped to the *Triticum aestivum* reference genome (PRJNA669381) using Bowtie (https://bowtie-bio.sourceforge.net/index.shtml) (Langmead et al., 2009). The reads were then specifically aligned to sRNA. The obtained reads were further aligned with noncoding RNA database to filter out rRNA, tRNA, and snoRNAs. The remaining unaligned and high-quality reads (Q30 ≥ 90%) were classified as known or novel miRNAs through a homology approach against Viridiplantae miRNAs from miRBase22 (miRBase) (Friedländer et al., 2012) using the NCBI-BLAST-2.2.304 with an e-value cutoff of e^-4^ and non-gapped alignment. Non-homologous sequences were analyzed using Mireap_0.22b, and those forming stem loop structures were considered as novel miRNAs. To eliminate the potential effect of *B. sorokiniana* miRNAs on wheat, all of the detected miRNAs were cross-referenced against *B. sorokiniana* genome.

### 2.4 Differential expression analysis of miRNA and its target prediction

Read counts for known and novel miRNAs were obtained to analyze their expression patterns. DE analysis was conducted using DESeq (version 2) (Anders and Huber, 2010), an R package with a threshold of log_2_ (fold change, FC) ≥1 and false discovery rate (FDR) ≤0.05. Read variations were normalized using DESeq2’s library normalization method, where size factors were calculated and each count was divided by its corresponding factor. The average normalized read counts of samples in each condition were utilized for differential gene expression calculation. For target prediction analysis, miRNAs with a copy number ≥5 were selected. These miRNA sequences were used as input along with wheat–*B. sorokiniana* transcriptome sequences (unpublished) to the miRanda tool (Griffiths-Jones et al., 2008), with the following default parameters: (1) using the strict alignment in the seed region (offset positions 2–8), preventing detection of target sites containing gaps or non-canonical base pairing in this region, (2) maximum expectation, and (3) target accessibility—allowed maximum energy to unpair the target site (UPE) 25. The DE miRNAs across eight sRNA-seq samples were clustered based on their log_2_FC values, with those displaying similar expression patterns grouped using R package Heatmap version 1.0. The associated target genes of DE miRNAs were analyzed for the GO and the KEGG enrichment to identify significantly enriched functional categories.

### 2.5 Annotation of miRNA-target genes, GO and KEGG pathway analysis

The identified target gene sequences were annotated with the BLAST2GO software (BLAST: Basic Local Alignment Search Tool (nih.gov), GO (http://www.geneontology.org/), KEGG (http://www.genome.jp/kegg/), Pfam (http://pfam.xfam.org/), InterPro (InterPro (ebi.ac.uk)), and Panther (pantherdb.org). Furthermore, the identified target genes were analyzed for GO (http://www.geneontology.org/) and KEGG (http://www.genome.jp/kegg/) (Kanehisa and Goto, 2000) enrichment analysis with FDR cutoff of 0.05. The networks of DE miRNAs along with their associated DE target genes were prepared using Cytoscape (v.3.10.2) (Shannon et al., 2003).

### 2.6 Validation of miRNAs and their target genes by qRT-PCR

The same samples used for sRNA-seq were also employed to validate miRNA expression and their corresponding target genes. RNA isolated from infected leaves was converted into cDNA using the Verso cDNA synthesis kit (Thermo Scientific) with miRNA-specific stem loop primers (Kramer, 2015). As per the protocol described by Varkonyi-Gasic et al. (2007), forward and universal reverse primers were designed for the selected nine miRNAs. The U6 small nuclear RNA (*U6 snRNA*) was the internal control gene for normalization. The correlation of miRNA expression in qRT-PCR (log_2_FC) and sRNA-seq was analyzed using the scatter plot. Based on the sequences of nine predicted target genes associated with the nine selected miRNAs, their specific qPCR primers were designed on GeneScript (Real Time PCR Primer Design - Real Time PCR Probe Design - GenScript). *β-actin* was used as an internal control gene. The qRT-PCR analysis of miRNAs and their corresponding target genes was performed using the Bio-Rad CFX96 system (Bio-Rad Laboratories, Inc., India) in the genomics laboratory of the Discovery Centre, ICAR-IARI, New Delhi, India. The list of primers used is given in **Table 1**.

**Table 1:** List of primers used in the study.

Sr. no.	miRNA/Gene Name	Primers
1.	miRNA	Stem loop primers
	tae-miR966a-3p	GTCGTATCCAGTGCAGGGTCCGAGGTATTCGCACTGGATACGAC**CGGTGG**
	os-miR5072	GTCGTATCCAGTGCAGGGTCCGAGGTATTCGCACTGGATACGAC**TGGCGA**
	ata-miR9863b-3p	GTCGTATCCAGTGCAGGGTCCGAGGTATTCGCACTGGATACGAC**GCTATT**
	ata-miR9863b-5p	GTCGTATCCAGTGCAGGGTCCGAGGTATTCGCACTGGATACGAC**GATGAG**
	xxx-m0030-3p	GTCGTATCCAGTGCAGGGTCCGAGGTATTCGCACTGGATACGAC**TCCTCA**
	xxx-m0005-3p	GTCGTATCCAGTGCAGGGTCCGAGGTATTCGCACTGGATACGAC**GTGCTC**
	xxx-m0007-5p	GTCGTATCCAGTGCAGGGTCCGAGGTATTCGCACTGGATACGAC**GGGAGG**
	xxx-m0009-5p	GTCGTATCCAGTGCAGGGTCCGAGGTATTCGCACTGGATACGAC**GCCCGG**
	xxx-m0031-5p	GTCGTATCCAGTGCAGGGTCCGAGGTATTCGCACTGGATACGAC**AACAGC**
	qPCR primers	
	tae-miR966a-3p	TTGAACATCCCAGAGCCACCG
	os-miR5072	TTCCCCAGCGGAGTCGC
	ata-miR9863b-3p	CGCGCGTGAGAAGGTAGATCATA
	ata-miR9863b-5p	GTTATGATCTGCTTCTCATC
	xxx-m0030-3p	CGCGCGCTTCGTGATCGATGT
	xxx-m0005-3p	ATCTTACCACGGCAGCTAGCG
	xxx-m0007-5p	ATCTTACCTCGTCGGTCGCGC
	xxx-m0009-5p	CACGCATCGCCTTCGAGAGAA
	xxx-m0031-5p	GCTGCTGCTGGTGTAGCTGTT
	Common reverse primer	CCAGTGCAGGGCCGAGTA
2.	Target Genes	
	UN(*XM_044510133.1*)-forward	AAGCTCTCCCACCTCAAGTC
	UN(*XM_044510133.1*)-reverse	CGATTTCAGAACGCGACAGT
	*NBS-ARC* forward	TAACAAGGAGGCAGGTCTGG
	*NBS-ARC* reverse	CAAGCTCTTCGTCACCCAAG
	*NBS-LRR* forward	GGCAACTGATTGAGCTGAGG
	*NBS-LRR* reverse	GGGACACCACCAAGGAACTA
	*RPP13L1* forward	GCTGCAGGTGTGGTCAGTTT
	*RPP13L1* reverse	GCACTTGTTGTTGGCTGCTG
	*CDK* forward	TTATGCCATTGTGCCGAGTG
	*CDK* reverse	TGTCGGCTGTACCTGAAGTT
	UN*(XM_044464186.1*)-forward	AGAGCCTGCAGTGAGTGATT
	UN(*XM_044464186.1*)-reverse	CCCTGGTGCTTGTTGTTGTT
	ATPB forward	GCACCAGGAGAAGGTAGGTT
	ATPB reverse	TGCCCTGTCTAGGGTTCTTG
	*PTK* forward	AGGAGTTCCAGGTGAGCTTC
	*PTK* reverse	ACATCTTGCGCCTCCTGATA
	TCF forward	TAGCAACCAGGCAGTACCAA
	TCF reverse	GCTGCTGGATCAAACTCTGG
	Reference genes	
	*U6* forward	GGGACATCCGATAAAATT
	*U6* reverse	TGGACCATTTCTCGATTT
	*β-ACTIN* forward	CAAATCATGTTTGAGACCTTCAAG
	*β-ACTIN* reverse	ACCAGAATCCAACACGATACCTG

## 3 Results

### 3.1 High-throughput sequencing and identification of known and novel miRNAs

**Figure 1:**
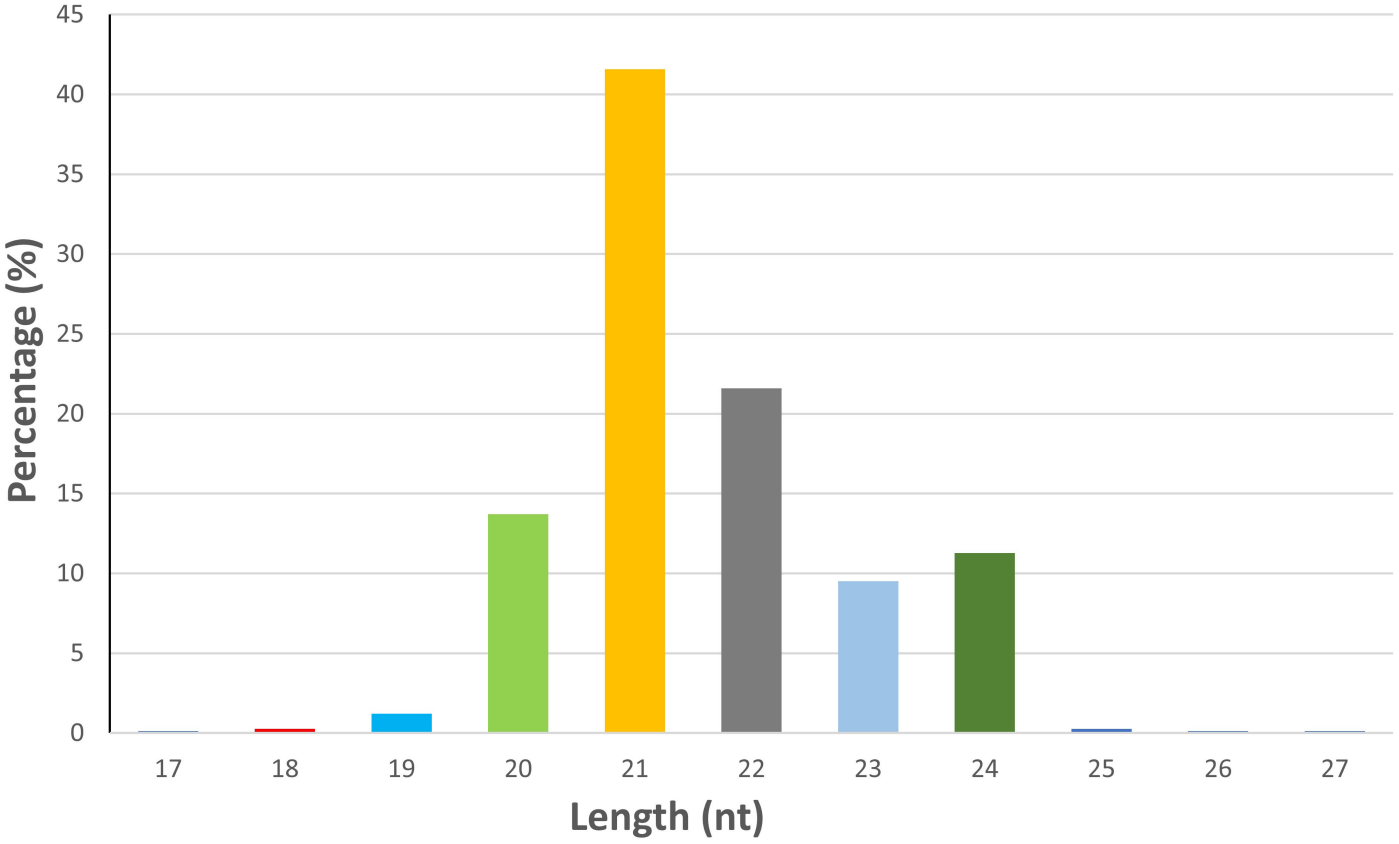
Relative frequency of length of identified miRNAs across all samples.

**Figure 2:**
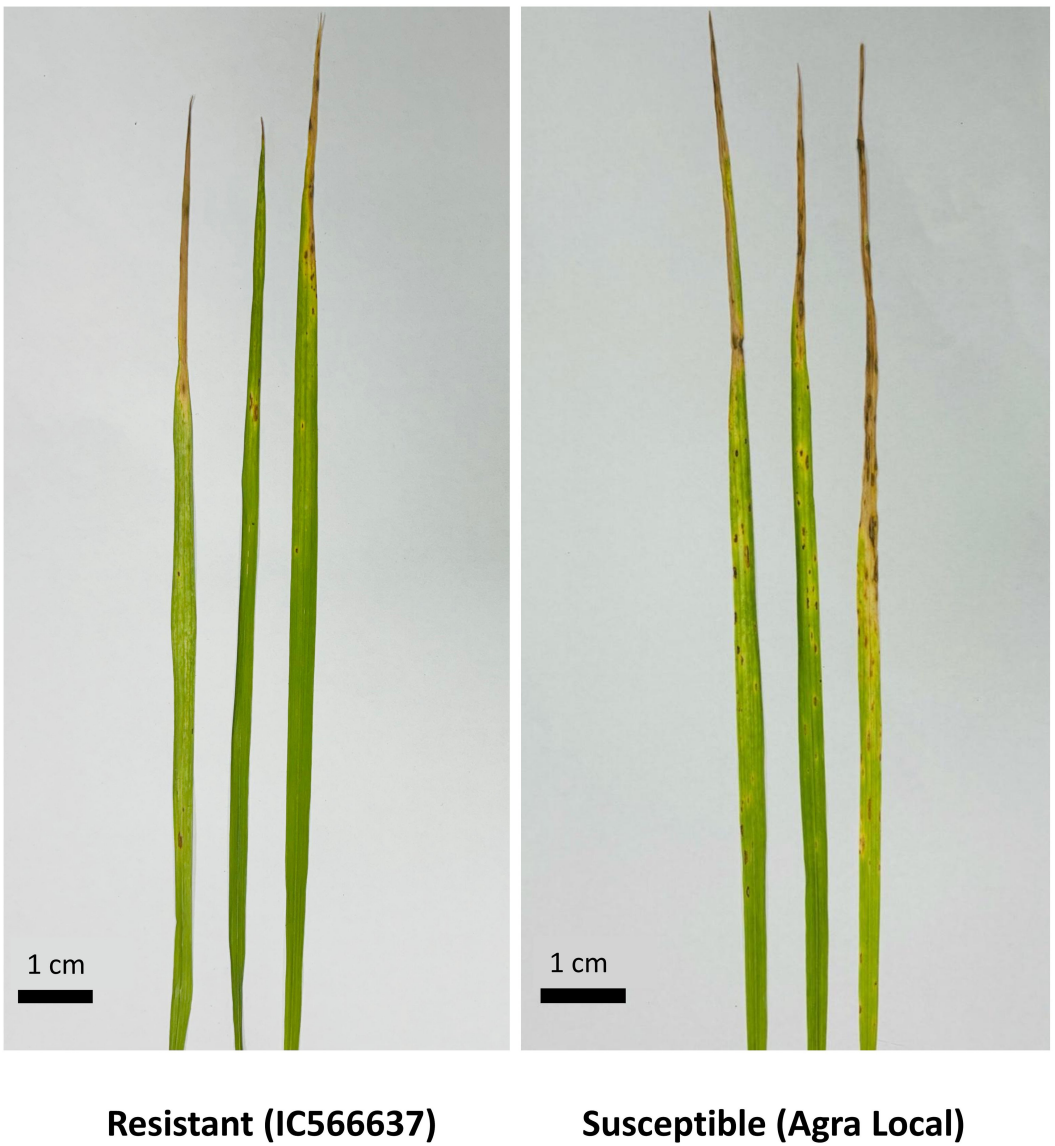
Visual symptoms on wheat leaves at 7 days post-inoculation with *Bipolaris sorokiniana*. Representative images show disease response in resistant (left) and susceptible (right) wheat lines.

An average of 19.73 million reads were generated as raw reads from the eight wheat samples comprising untreated and *B. sorokiniana-*inoculated wheat leaves. This data has been uploaded in the Sequence Read Archive (SRA) database of NCBI with Bioproject number PRJNA944096. After stringent quality filtering and removal of low-quality reads, adapters, and sequences shorter than 16 nt, an average of 12.2 million high-quality reads (Q30 ≥ 90%) with a read length between 16 and 40 nt were retained for downstream analysis (**Supplementary Table S1**). A total of 736 miRNAs were identified across all samples, including 418 known and 318 novel miRNAs (**Supplementary Table S2**). The majority of the identified miRNAs were 21 nt (41.57%) in length, followed by 22 nt (21.60%), 20 nt (13.72%), and 24 nt (11.27%) (**Figure 1**). The symptomatic response of the resistant genotype IC566637 and suscpetible variety Agra local at 4 days post-inoculation is shown in **Figure 2**.

### 3.2 MicroRNA expression profiles upon *B. sorokiniana* infection

**Figure 3:**
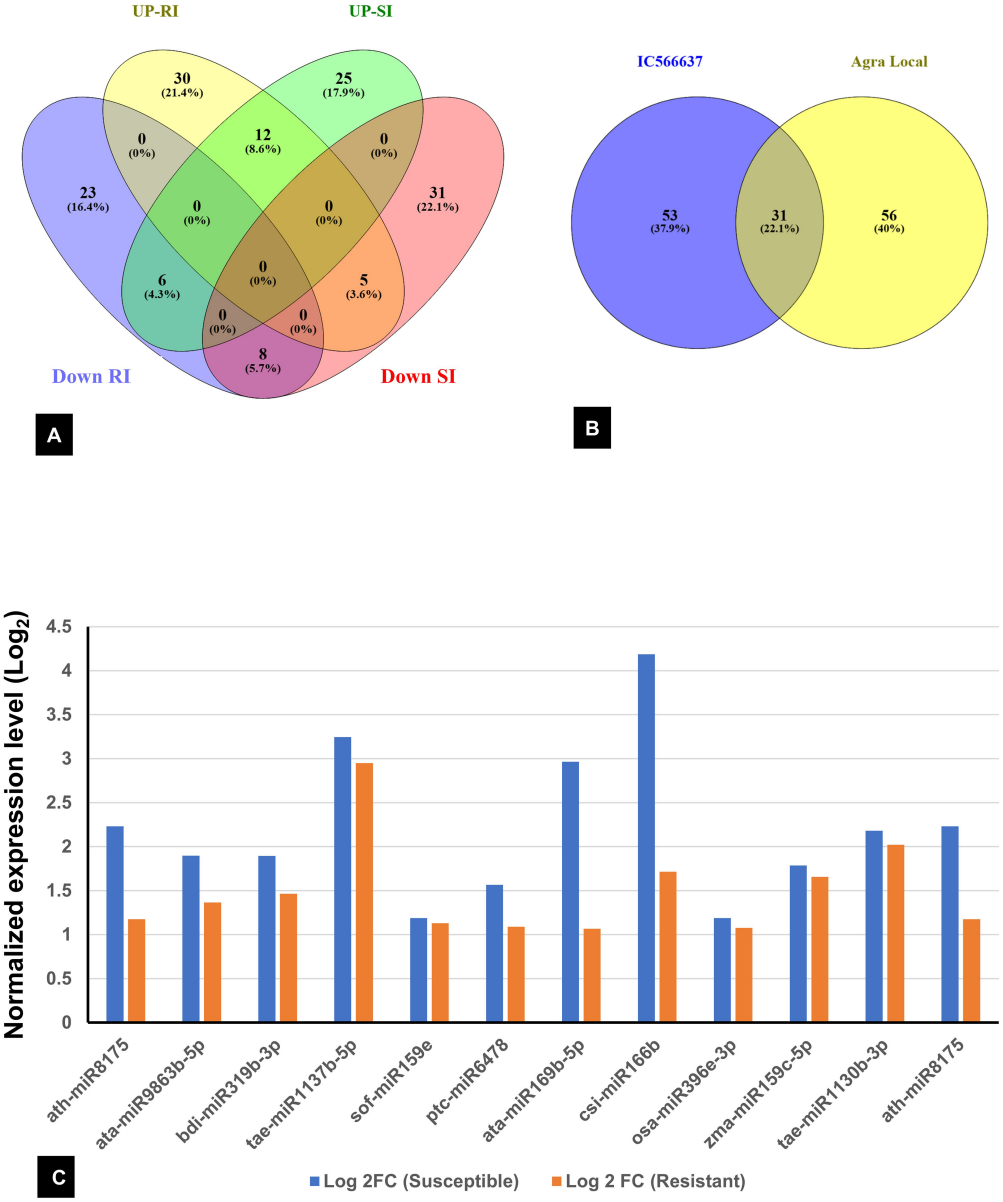
**(A)** Venn diagram showing common and exclusive differentially expressed (DE) miRNAs across all samples. **(B)** Total DE miRNA in resistant IC566637 and susceptible Agra Local; UP-RI and DR-RI: upregulated and downregulated in resistant IC566637 inoculated with *B. sorokiniana*, respectively; UP-SI and DOWN-SI: upregulated and downregulated in susceptible Agra Local inoculated with *B. sorokiniana*, respectively. **(C)** List of miRNAs up-regulated in both IC566637 and Agra Local.

**Figure 4:**
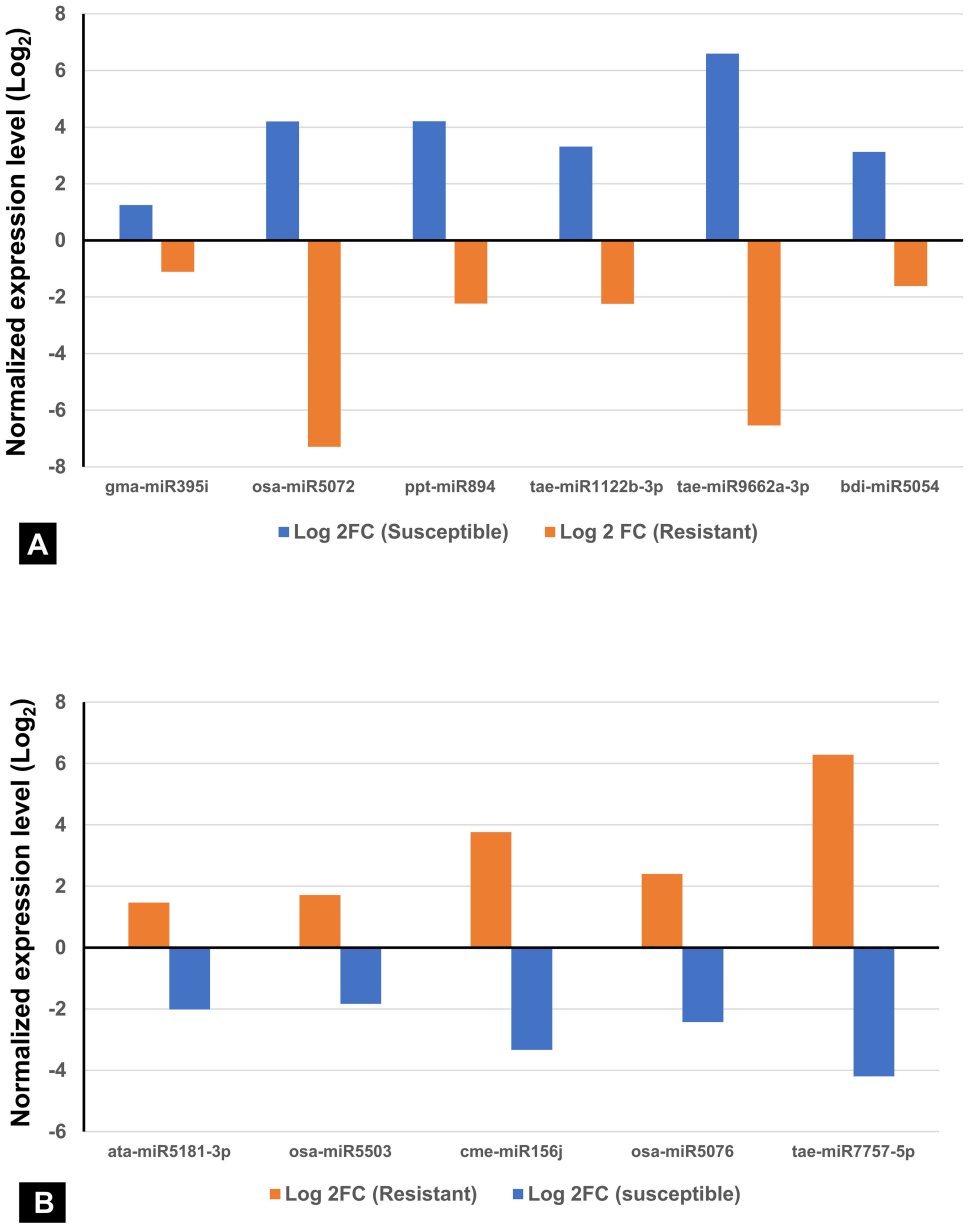
Contrasting expression patterns of miRNAs in spot-blotch-resistant wheat genotype (IC566637) and susceptible variety (Agra Local) after *B. sorokiniana* infection. **(A)** miRNA upregulated in susceptible and downregulated in resistant lines. **(B)** miRNA upregulated in resistant and downregulated in susceptible lines.

Among the 736 identified miRNAs across the control and treated groups of the two wheat lines, 140 were DE miRNAs in response to infection. The number of DE miRNAs was slightly higher in SI than in RI (**Supplementary Table S3**). Specifically, 44 miRNAs were upregulated and 43 downregulated in SI, whereas 37 were upregulated and 47 downregulated in RI. A total of 31 DE miRNAs were common between RI and SI, indicating that some miRNA-mediated regulatory responses are common to both resistant and susceptible backgrounds. However, genotype-specific expression patterns were also evident: 30 and 23 miRNAs were uniquely up- and downregulated in RI, while 25 and 31 were unique to SI, respectively. A total of 12 miRNAs were found to be upregulated in both the RI and SI, with all exhibiting higher log_2_FC values in SI, suggesting stronger transcriptional activation of these miRNAs under susceptible background (**Figures 3A, B**). Interestingly, several miRNAs showed contrasting expression in RI and SI; tae-miR1122b-3p, tae-miR9662a-3p, bdi-miR5054, gma-miR395i, osa-miR5072, and ppt-miR894 were downregulated in RI and were found to be upregulated in SI (**Figure 4A**). Conversely, ata-miR5181-3p, osa-miR5503, cme-miR156j, osa-miR5076, and tae-miR7757-5p were found to be upregulated in RI and downregulated in SI (**Figure 4B**).

### 3.3 Prediction of miRNA targets

For the 140 DE miRNAs, a total of 894 putative target genes were predicted and functionally annotated based on GO, KEGG, Panther, InterPro, and Pfam (**Supplementary Table S4**). The identified target genes represented a broad range of functional categories. Majority of the identified target genes were associated with cellular signaling processes such as protein kinases, including serine/threonine protein kinases and calcium-transporting ATPases. Several targets were involved in plant-defense-associated functions, including xylanase inhibitors (TAXI-IV and XI-IB), beta-1,3-glucanase (Glb3), peroxidases (Prx111-A and Prx112-M), chitinases, and resistance-related proteins such as RPP13-like, Nucleotide-binding Apaf-1 R proteins CED-4 (NB-ARC), and LRR proteins. In addition, a number of target genes encoded transcription factors, including WRKY10 and zinc finger proteins (ZFPs), which are often linked with regulatory control of gene expression. Several transporter proteins were also identified among the targets, such as members of the sulfite exporter TauE/SafE family, putative zinc transporter (ZIP1), and ABC transporters. Furthermore, multiple transferase enzymes were predicted, including acyl transferases (acT1) and ubiquitin transferases (RING-type E3).

### 3.4 GO and KEGG pathway analysis of target genes

**Figure 5:**
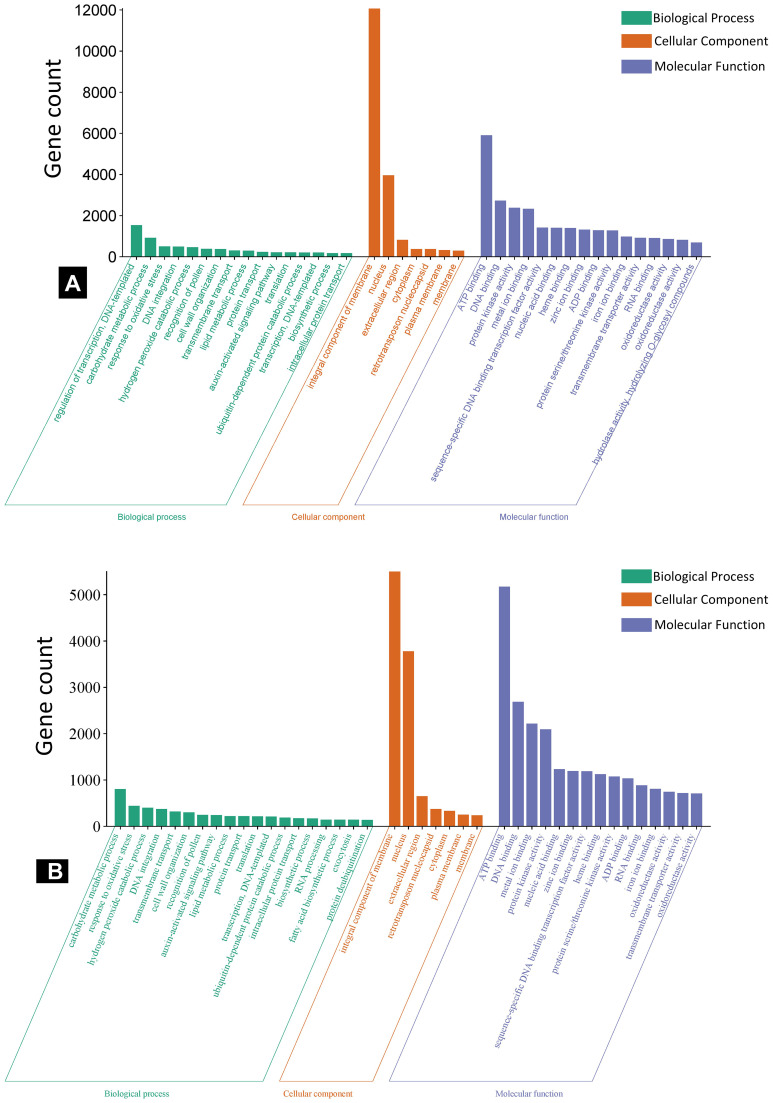
WEGO plots showing GO enrichment analysis of miRNA target genes in spot-blotch-resistant wheat genotype IC566637 **(A)** and susceptible variety Agra Local **(B)** infected with *B. sorokiniana*.

The GO enrichment analysis of predicted target genes was grouped into biological process, cellular components, and molecular functions (**Figure 5**). For the specific DE miRNAs in IC566637, the predicted target genes participated in 144, 114, and 12 GO terms for biological process, molecular function, and cellular component, respectively (**Supplementary Table S5**; **Figure 5A**). However, the predicted target genes for DE miRNAs specific in Agra Local participated in 92, 104, and five GO terms for biological process, molecular function, and cellular component, respectively (**Supplementary Table S6**; **Figure 5B**). The KEGG pathway enrichment analysis indicated that the predicted target genes were mainly involved in 15 significantly enriched pathways (**Table 2**). In the resistant genotype IC566637, the most enriched pathways included biosynthesis of secondary metabolites, valine, leucine, and isoleucine biosynthesis, diterpenoid biosynthesis, fatty acid metabolism, and the MAPK signaling pathway. In the susceptible genotype Agra Local, the enriched pathways included linoleic acid metabolism, phenylpropanoid biosynthesis, glucosinolate biosynthesis, cysteine and methionine metabolism, valine, leucine, and isoleucine metabolism, plant–pathogen interaction, and ubiquinone and other terpenoid–quinone biosynthesis.

**Table 2:** KEGG pathway enrichment analysis of predicted target genes associated with DE miRNAs in spot-blotch-resistant wheat genotype IC566637 and susceptible variety Agra Local after *B. sorokiniana* infection.

Genotype	Pathway	Pathway ID	Enrichment FDR	Fold enrichment	Number of genes
IC566637	Biosynthesis of secondary metabolites	taes01110	5.36E-08	5.16975484	24
	Valine leucine and isoleucine biosynthesis	taes00290	8.67E-05	23.02890792	5
	ABC transporters	taes02010	8.67E-05	9.211563169	8
	Plant hormone signal transduction	taes04075	0.000131123	4.068711647	15
	Diterpenoid biosynthesis	taes00904	0.000299579	10.96614663	6
	Fatty acid metabolism	taes01212	0.000299579	7.058669095	8
	MAPK signaling pathway	taes04016	0.000441066	4.995424712	12
Agra Local	Linoleic acid metabolism	taes00591	0.001447965	21.54346955	4
	Phenylpropanoid biosynthesis	taes00940	0.001447965	4.113563341	13
	Glucosinolate biosynthesis	taes00966	0.006520616	22.70798142	3
	Cysteine and methionine metabolism	taes00270	0.007427031	5.092092803	7
	Valine leucine and isoleucine biosynthesis	taes00290	0.0079465	3.895805158	3
	Valine leucine and isoleucine degradation	taes00280	0.011115878	16.80390625	4
	Plant–pathogen interaction	taes04626	0.053660481	2.82079961	8
	Ubiquinone and other terpenoid-quinone biosynthesis	taes00130	0.071282116	5.154572469	3

### 3.5 Clustering analysis of DE miRNAs

**Figure 6:**
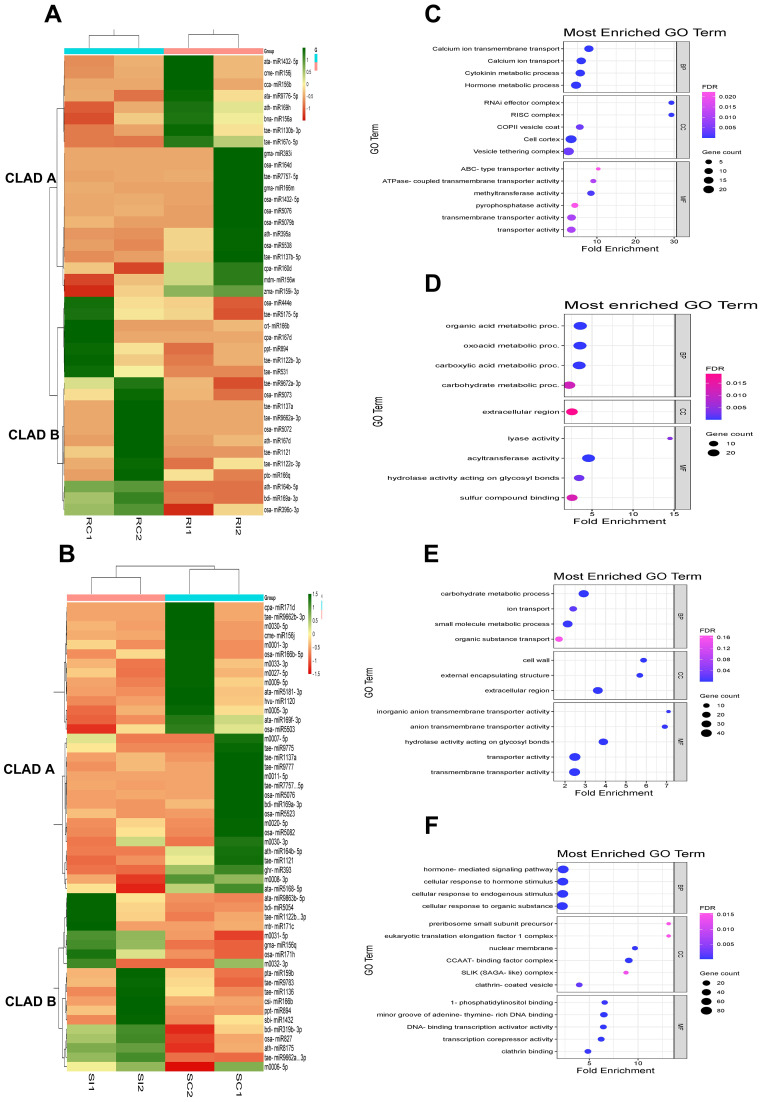
Clustering of expression profiles of DE miRNAs and GO enrichment analysis of their target genes in **(A)** resistant IC566637 and **(B)** susceptible Agra Local infected with *B. sorokiniana*. **(C)** GO enrichment analysis for target genes associated with DE miRNAs in IC566637 in clade A and **(D)** clade B. **(E)** GO enrichment analysis for target genes associated with DE miRNAs in Agra Local in clade A and **(F)** clade **(B)** The GO enrichment analysis of DE target genes was analyzed for DE miRNAs cladewise, with *p*-value cutoff of 0.05.

Hierarchical clustering and heat map analysis revealed distinct expression patterns with upregulated (green) and downregulated (red) miRNAs in IC566637 and Agra Local, forming two separate clades that indicate expression heterogeneity (**Figure 6**). For IC566637, clade A contained upregulated and clade B with downregulated miRNAs in RI compared to RC samples (**Figure 6A**). However, in Agra Local, clade A shows downregulated and clade B with highly upregulated miRNAs in SI compared to SC samples (**Figure 6B**). The predicted DE target genes of miRNA for IC566637 in clade A were predominantly enriched in GO terms with transporter activity (calcium ion transmembrane transport, ABC transporters, ATPase coupled transporter activity, transmembrane transporters, and methyltransferase), cytokinin and hormone metabolic process, RNAi effector complex, RISC complex, and COPII vesicle coat and vesicle tethering complex (**Figure 6C**). However, the predicted DE target genes in IC566637 were mainly enriched with organic acid, carbohydrate, carboxylic acid metabolic process, extracellular region, lyase and acyltransferase activity, hydrolase activity acting on glycosyl bonds, and sulfur compound binding (**Figure 6D**). The predicted DE target genes of miRNA in clade A of Agra Local were mainly enriched in carbohydrate metabolic process, ion transport, small molecule metabolic process, organic substance transport, cell wall, external encapsulating structure, extracellular region, and transporter activity (**Figure 6E**). For miRNAs in Agra Local (clade B), the DE-predicted target genes were mainly enriched in hormone-mediated signaling pathway, cellular response to hormone stimulus, endogenous stimulus and organic substance, pre-ribosome small subunit precursor, eukaryotic translation elongation factor 1 complex, nuclear membrane, CCAAT-binding factor complex, SLIK (SAGA-like) complex, clathrin-coated vesicle, 1-phosphatidylinositol binding, minor groove of adenine–thymine-rich DNA binding, DNA-binding transcription activator activity, and transcription corepressor activity (**Figure 6F**). The results suggest that miRNAs exhibiting varying expression levels could have unique functions in regulating their target genes engaged in different biological pathways during *B. sorokiniana* and wheat interaction.

### 3.6 Validation of miRNAs and their target genes by qRT−PCR

**Figure 7:**
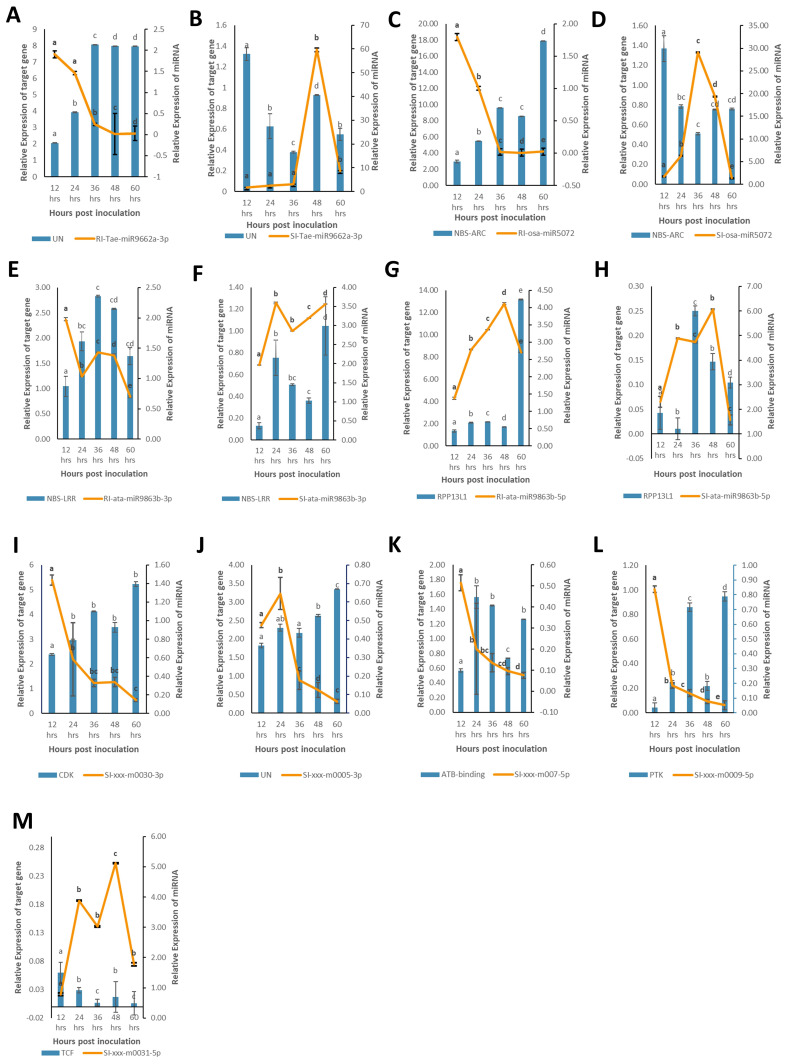
Expression profiles of nine selected miRNAs (line charts) and their associated nine target genes (bar charts) in resistant IC566637 and susceptible Agra Local infected with *B. sorokiniana* through qRT-PCR analysis. The relative abundance of miRNAs and its associated target gene is depicted on the right and left y-axis, respectively. Each bar indicates the mean values of three biological replicates ± standard error (*n* = 3). Based on *post-hoc* Tukey’s HSD test (*p*-value < 0.05), significant difference is represented by various lowercase letters. Subpanels **(A–H)** represent the expression profiles of conserved miRNAs and their target genes in resistant and susceptible lines, respectively. Subpanels **(I–M)** show the expression profiles of novel miRNAs in the susceptible line. UN, uncharacterized; NB-ARC, nucleotide-binding ARC domain encoding gene; NB-LRR, nucleotide-binding leucine-rich repeat domain encoding gene; *RPP13L1*, putative disease resistance *RPP-13* like protein-1; *CDK*, cyclin-dependent serine–threonine protein kinase; *PTK*, protein kinase; *TCF*, transcription cofactor.

**Figure 8:**
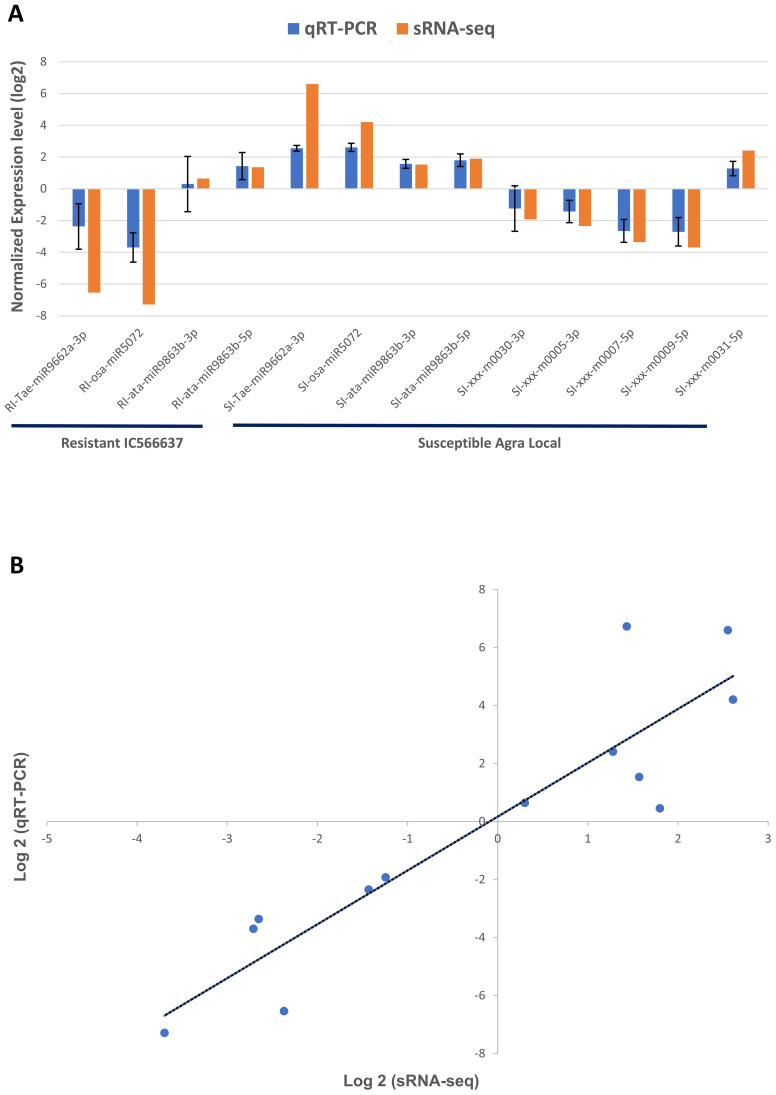
Log_2_FC values of DE miRNAs in **(A)** qRT-PCR and small-RNA seq and their **(B)** correlation analysis by scatter plot. The error bar indicates the mean ± standard error values of log_2_ fold change for the three biological replicates for the miRNAs validated through qRT-PCR.

**Figure 9:**
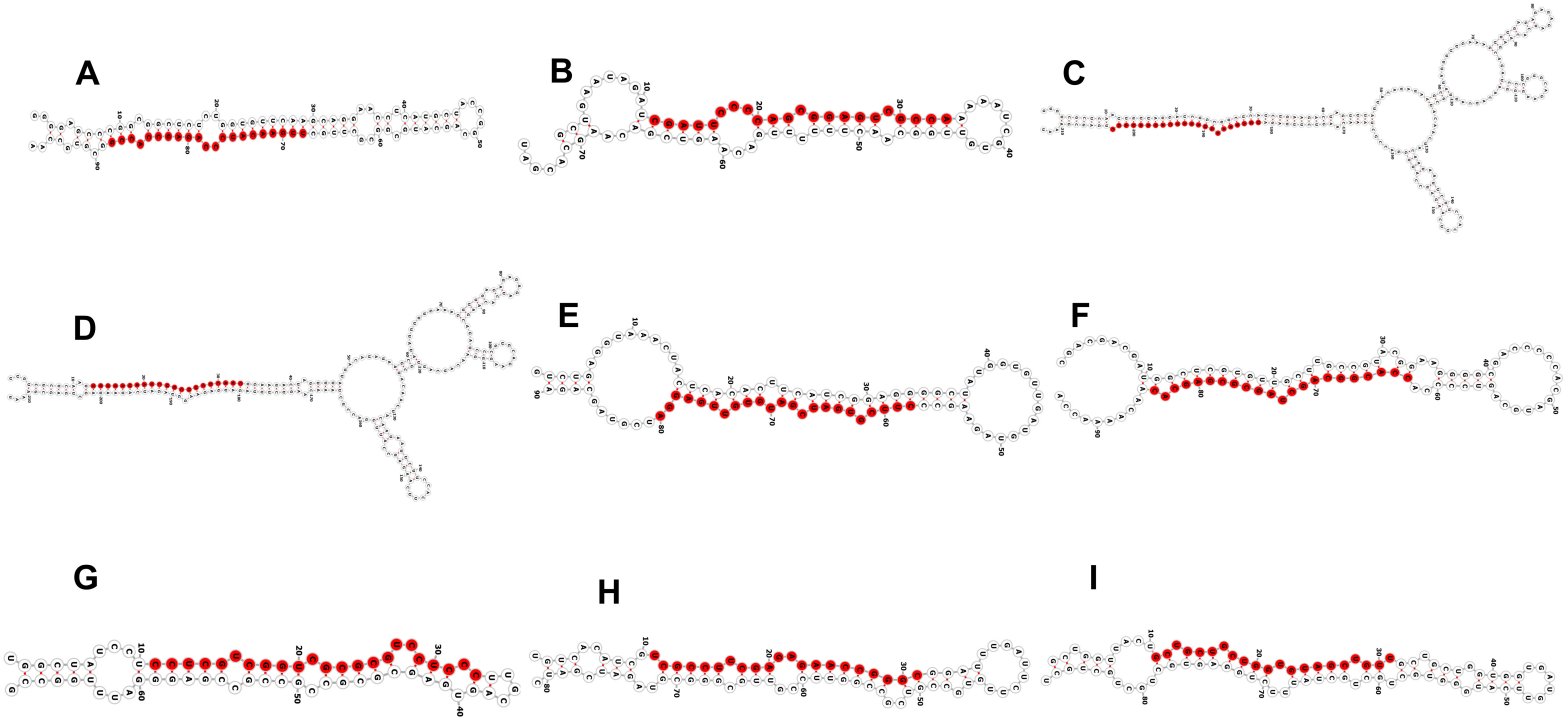
Stem loop structures of known and novel differentially expressed miRNAs validated through stem loop-qRT-PCR. **(A)** tae-miR9662a-3p, **(B)** osa-miR5072, **(C)** ata-miR9863b-3p, **(D)** ata-miR9863b-5p, **(E)** novel xxx-m0030-3p (Chr7A: 234026622.234926533), **(F)** novel xxx-m0005-3p (Chr1A: 162410073.162409980), **(G)** novel xxx-m0007-5p (Chr3B: 829583.829643), **(H)** novel xxx-m0009-5p (Chr3A: 71549420.71549340), and **(I)** novel xxx-m0031-5p (Chr6B: 676244915.676244828). The mature sequence of the respective miRNA is represented in red. The stem loop structures are visualized using RNA fold server (RNAfold web server).

In this study, we evaluated the expression of nine miRNAs selected based on lowest *p*-value and highly variable expression (either significantly upregulated or downregulated) and their corresponding target genes associated with defense response against *B. sorokiniana* in IC566637 and Agra Local. Among these, the expression of four known miRNAs (tae-miR9662a-3p, osa-miR5072, ata-miR9863b-3p, and ata-miR9863b-5p) and five novel miRNAs (xxx-m0030-3p, xxx-m0005-3p, xxx-m0007-5p, xxx-m0009-5p, and xxx-m0031-5p), respectively, was assessed at five different (12, 24, 36, 48, and 60 h) time points using qRT-PCR (**Figure 7**). The expression of these miRNAs varied considerably across different time points. Notably, three known miRNAs (tae-miR9662a-3p, osa-miR5072, and ata-miR9863b-3p) exhibited contrasting expression patterns: they were downregulated in IC566637 and upregulated in Agra Local. For instance, the expression of tae-miR9662a-3p reduced considerably in RI, which corresponded with the upregulation of its target gene (XM_044510133.1: gene involved in defense response to fungus) that reached 8.06-fold increase at 36 h. Conversely, in SI, the expression of tae-miR9662a-3p drastically increased to 59.74-fold at 48 h, while its target gene was downregulated. Similarly, osa-miR5072 and ata-miR9863b-3p were found to be downregulated in RI and upregulated in SI. The osa-miR5072 exhibited its highest expression in SI at 29.12 times that of the control, while ata-miR9863b-3p reached 3.59 times that of the control in SI. Correspondingly, the target genes for osa-miR5072 (NB-ARC protein) and ata-miR9863b-3p (NB-LRR protein) were downregulated in SI while being upregulated in RI, reaching up to 17.89-fold at 24 h and 2.83-fold at 36 h, respectively. The expression of ata-miR9863b-5p was significantly upregulated in both RI and SI, reaching up to 4.11-fold and 6.07-fold at 48 h, respectively. Notably, its target gene, putative disease resistance (*RPP13*-like protein 1), showed differential regulation. In RI, the gene was positively regulated by the miRNA with an upregulation of 13.19-fold compared to the control. Conversely, in SI, a negative regulation was observed, where the target gene was found to be downregulated. Among the predicted novel miRNAs evaluated for expression, four miRNAs—xxx-m0030-3p, xxx-m0005-3p, xxx-m0007-5p, and xxx-m0009-5p—were found to be downregulated in SI. Contrarily, the corresponding target genes for xxx-m0030-3p (cyclin-dependent protein serine/threonine kinase), xxx-m0005-3p (peroxidase), and xxx-m0007-5p (ATP-binding, involved in defense response) were up-regulated in SI, rising to 5.23-fold at 60 h, 3.3-fold at 60 h, and 1.56-fold at 24 h, respectively, showing a negative regulatory relationship. However, for the target gene for xxx-m0009-5p (protein kinase), a positive regulation was observed, where the target gene was also downregulated in SI. The other miRNA xxx-m0031-5p was significantly upregulated in SI, rising to 5.12-fold compared to the control at 48 h, with corresponding downregulation of its target gene (transcription cofactor). The expression profile of these DE miRNAs in qRT-PCR showed a strong correlation (*R*^2^ = 0.915) with sRNA-seq data (**Figure 8**). The stem loop structures of validated miRNAs were depicted (**Figure 9**).

## 4 Discussion

Spot blotch is among the most devastating diseases of wheat, triggering significant economic losses, which are expected to be aggravated in climate change scenarios. The hemibiotrophic lifestyle and high population diversity of the pathogen, coupled with the lack of qualitative resistance in wheat, pose significant challenges in managing spot blotch disease. In plants, during pathogen infection, variation in miRNA level modulates gene expression, which regulates molecular pathways, including signals for biotic stress responses (Anders and Huber, 2010; Li et al., 2010; Friedländer et al., 2012). To investigate miRNA-mediated regulation in response to *B. sorokiniana*, sRNA-seq analysis was conducted on the resistant genotype IC566637 and susceptible variety Agra Local. In this study, 726 miRNAs with a length of 20–24 nt were identified from the sRNA-seq data (**Figure 1**), consistent with the earlier reports from Su et al. (2022) and Jin et al. (2024). The correlation between the qRT-PCR and sRNA-seq experiments was *R*^2^ = 0.915, indicating a strong correlation and, thus, consistency and reliability of the high-throughput sequencing for the discovery of novel and DE miRNAs. Therefore, the miRNA seq data from this study will enrich the wheat miRNA library.

**Figure 10:**
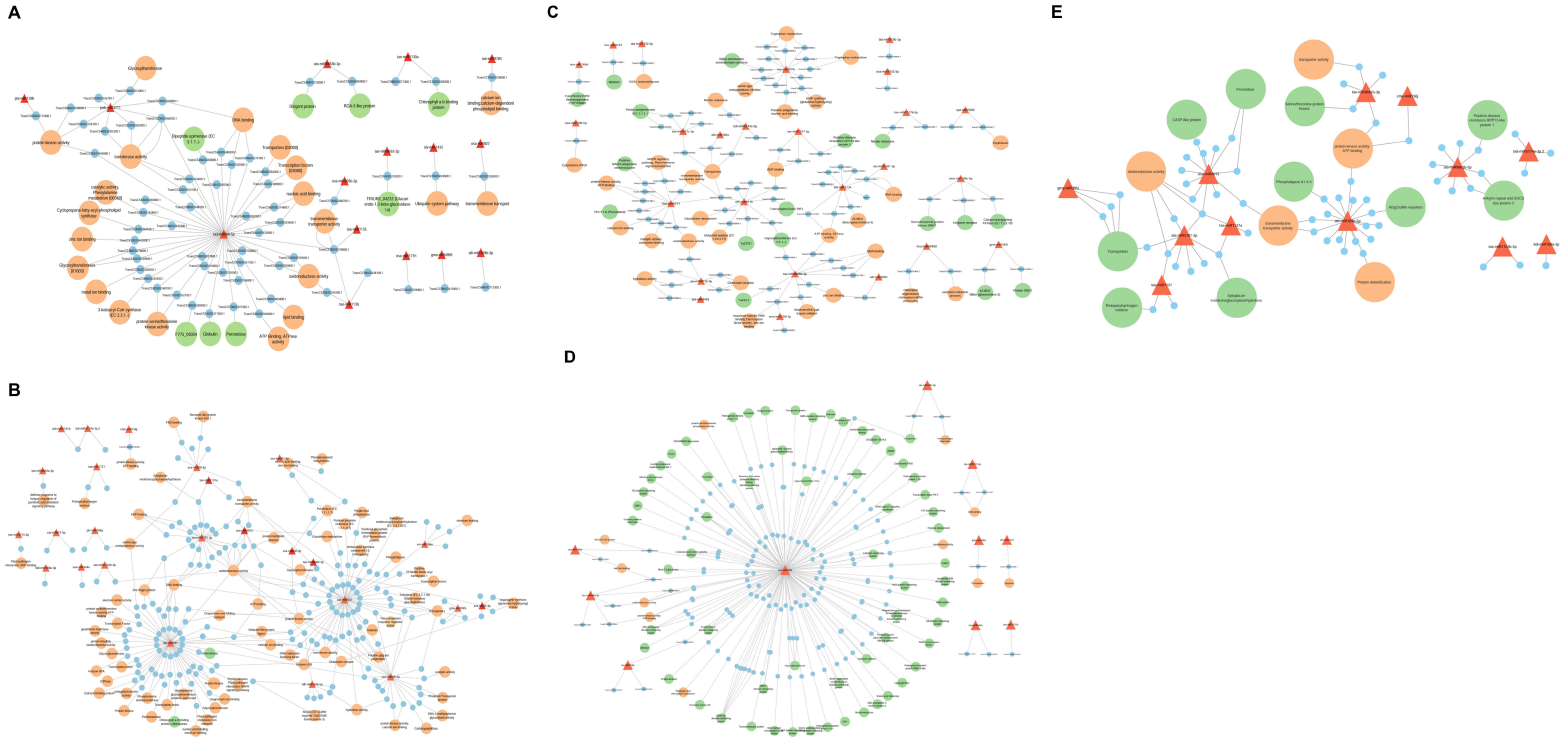
Regulatory networks of DE miRNAs and their corresponding target genes in resistant genotype IC566637 and susceptible variety Agra Local after *B. sorokiniana* infection. **(A)** DE miRNAs upregulated in Agra Local. **(B)** DE miRNAs downregulated in Agra Local. **(C)** DE miRNAs upregulated in IC566637. **(D)** DE miRNAs downregulated in IC566637. **(E)** Common DE miRNAs in IC566637 and Agra Local.

Plant miRNAs play a crucial role in regulating metabolic pathways by targeting associated genes. In this study, among the DE miRNA target genes identified in IC566637, 24 were significantly enriched in secondary metabolite biosynthesis, 15 in plant hormone signal transduction, and 12 in the MAPK signaling pathway. However, for the target genes from Agra Local, 13 genes showed significant enrichment in phenylpropanoid biosynthesis pathway, eight in plant–pathogen interaction pathway, glucosinolate and ubiquinone, and other terpenoid-quinone biosynthesis, indicating that these genes modulated by the DE miRNAs possibly play a vital role in wheat’s response to *B. sorokiniana* infection. Previous experimental studies in wheat have demonstrated that hormone-mediated signaling components, particularly those linked to salicylic acid (SA), jasmonic acid (JA), and ethylene (ET) pathways, play key roles in the wheat–*B. sorokiniana* interaction (Eisa et al., 2013; Sahu et al., 2016). These reports highlighted that the differential activation of SA- and JA-mediated responses contributes to variation in resistance and susceptibility among wheat genotypes. Consistent with these findings, the enrichment of hormone-related pathways in our dataset suggests the engagement of phytohormone signaling as part of the defense response to spot blotch. Notably, our results further extend this understanding by revealing that several of these hormone-associated genes are potential targets of DE miRNAs, indicating a possible regulatory layer through which miRNAs fine-tune hormonal signaling and downstream defense mechanisms. Studies have also shown that these miRNA families regulate defense-related genes directly or indirectly by synthesizing small interfering RNAs (siRNAs) involved in gene silencing. The miRNAs can regulate their target genes, which can be attributed to the plant’s response to disease either directly or indirectly. In our study, the regulatory network analysis of DE miRNAs and their target genes (**Figure 10**) identified ata-miR9863b-3p, ata-miR9863b-5p, tae-miR5175-5p, tae-miR9662a-3p, cme-miR156j, and zma-miR159c-5p that directly regulate the plant immune receptor genes (NB-LRR) or genes that are directly involved in host–pathogen interactions. Similar results have been reported by Liu et al. (2014), where miR9863a and miR9863b were found to target and repress the expression of many NB-LRR genes in wheat and barley. Several other DE miRNAs and their corresponding target genes were found to be indirectly involved in plant immunity, mainly bdi-miR531, tae-miR531, ata-miR5181-3p, osa-miR1848, and novel miRNA xxx-m0064-5p, which were discovered to have complex and diversified target genes (**Figure 10**). Some of these target genes of DE miRNAs have pleiotropic effects regulating plant growth and development, which, in turn, influences disease response (Islam et al., 2018; Šečić et al., 2021).

MicroRNAs regulate their target genes either positively (Su et al., 2019) or negatively (Schwab et al., 2005), as also observed in our study. For instance, the regulation modes of ata-miR9863b-5p on its target gene expression were different in the two wheat lines, with a positive and negative regulatory role in RI and SI, respectively. This irregular expression pattern of miRNA–target genes has also been observed in rice and sugarcane (Su et al., 2019, 2022) in response to rice black-streaked dwarf virus, sorghum mosaic virus, and sugarcane smut. Both the novel miRNA xxx-m0009-5p and its target gene encoding a protein kinase were downregulated in SI, suggesting a possible positive regulatory relationship between the miRNA and its target gene. The expression pattern of tae-miR9662a-3p, osa-miR5072, and ata-miR9863b-3p exhibited contrasting behavior in the two wheat lines, showing downregulation in resistant genotype and upregulation in the susceptible variety. Notably, their corresponding target genes *NB-ARC* (*XM_044482261.1*) and *NB-LRR* (*XM_044591884.1*), involved in defense response to fungus, also demonstrated contrasting expression, being upregulated in RI and downregulated in SI. Interestingly, all of these target genes showed significant upregulation in RI and conversely downregulation in SI, both at 36 h post-infection, indicating it as the critical stage of defense against *B. sorokiniana* in wheat. Furthermore, *NB-LRR* genes are among the most widely investigated disease resistance gene families in plants (Jones and Jones, 1997). Liu et al. (2014) documented that miR9863 leads to the silencing of *NB-LRR* genes in wheat and barley, significantly reducing gene transcripts at 36 h post-inoculation. A negative regulation in the expression of *NB-LRR* transcripts was associated with the expression of miR482, thus conferring resistance to cotton against *Verticillium dahliae* (Zhu et al., 2013). Similar responses are reported for tomato and poplar for infection against *Cytospora chrysosperma* and *Colletotrichum gloeosporioides* by targeting *NB-LRR* transcripts (Ouyang et al., 2014; Su et al., 2018). Furthermore, poplar miR472a and *Arabidopsis* miR472 also trigger the synthesis of secondary phasiRNAs for systemic *NB-LRR* silencing (Boccara et al., 2015). The expression of ata-miR9863b-5p was found to increase more fold in SI (up to 6.07-fold) than RI (up to 4.11-fold) at 48 h. Consequently, its target gene (*RPP13L1*) was upregulated in RI and downregulated in SI. Moreover, it should be noted that the expression of *RPP13L1* increased to a higher fold in earlier time points after infection. Thus, this differential regulation of expression could be attributed to miRNA expression abundance in SI wherein the higher level of miRNA expression increased the likelihood of target silencing. Conversely, in RI, rapid target transcript abundance diluted miRNA efficacy. The key determinant of resistance or susceptibility lies in rapid signal recognition and the efficacy of host’s defense response following pathogen invasion (Su et al., 2022). Arvey et al. (2010) have proved that the abundance of target mRNA dilutes the activity of miRNA and siRNA. The *RPP13*-like genes are a part of the *NB-LRR* superfamily and are significantly upregulated in wheat when challenged with *Puccinia striiformis* f. sp. *tritici* and *Blumeria graminis* f. sp. *tritici*, causing stripe rust and powdery mildew diseases, respectively (Zhang et al., 2021).

In plant species, protein kinases are key signaling molecules that regulate protein activity through reversible phosphorylation and also play crucial roles in host–pathogen interactions (Cohen, 2000; Zhu et al., 2017; Goyal et al., 2018; Jiang et al., 2021). They are involved in both compatible (susceptibility) and incompatible (resistance) interactions. In this study, the expression of xxx-m0030-3p was downregulated in SI, accompanied by the upregulation of its target gene XM_044508546.1, which encodes a cyclin-dependent serine/threonine protein kinase (*CDK*). The results indicate the involvement of *CDK* in wheat’s susceptibility to *B. sorokiniana* infection. In *Arabidopsis*, *CDK* proteins such as *CDK9*-like proteins, *CDKC1*, and *CDKC2* play crucial roles in infection and increase susceptibility to the cauliflower mosaic virus (CaMV) (Cui et al., 2007). A MAPK in wheat (*TaMAPK4*), a positive regulator of defense in wheat–*P. striiformis* f. sp. *tritici* infection, is targeted by tae-miR164 (Wang et al., 2018).

## 5 Conclusion

In the present research, a comprehensive miRNA database was developed to analyze wheat’s response to *B. sorokiniana* infection. A total of 418 known and 318 novel miRNAs were identified, among which 140 DE miRNAs were predicted to be involved in wheat’s response to *B. sorokiniana*, targeting 894 genes. Following infection, the number of DE miRNAs was slightly higher in the susceptible variety Agra Local (87) than the resistant genotype IC566637 (84). KEGG enrichment analysis revealed that the predicted targets of these DE miRNAs were predominantly associated with defense pathways, including biosynthesis of secondary metabolites, ABC transporters, plant hormone signal transduction, MAPK signaling, and plant–pathogen interaction. Regulatory network analysis highlighted key miRNAs, particularly from miR9863, miR156, and miR159 families, specifically targeting *NB-LRR*s or kinase genes. Furthermore, the expression profiles of four known miRNAs (tae-miR9662a-3p, osa-miR5072, ata-miR9863b-3p, and ata-miR9863b-5p) and five novel miRNAs (xxx-m0030-3p, xxx-m0005-3p, xxx-m0007-5p, xxx-m0009-5p, and xxx-m0031-5p), along with their corresponding target genes, were evaluated with qRT-PCR. The study identified the expression of putative disease resistance *RPP13*-like protein and other uncharacterized *NB-LRR*/protein kinase genes known widely in plant–pathogen interactions. Overall, this work represents an initial broad-scale survey of wheat miRNA responses to *B. sorokiniana*. While pooling of time points allowed the generation of an exploratory dataset, the parallel qRT-PCR analysis of individual time point samples provided temporal insights into the expression of key miRNAs. Future studies using sequencing at individual time points will be essential to capture fine-scale, transient regulatory dynamics. Moreover, exploring the role of pathogen-derived sRNAs and their possible cross-kingdom effects on wheat defense responses will provide a more comprehensive understanding of this interaction. Overall, the present study enhances the wheat miRNA library and serves as an important foundational work in understanding miRNA-mediated regulation in wheat during *B. sorokiniana* infection, providing potential candidate genes to develop spot blotch-resistant wheat varieties through resistance breeding and gene editing.

## Data Availability

The datasets presented in this study can be found in online repositories. The names of the repository/repositories and accession number(s) can be found in the article/**Supplementary Material**.
